# Methodology and Significance of Microsensor-based Oxygen Mapping in Plant Seeds – an Overview

**DOI:** 10.3390/s90503218

**Published:** 2009-04-27

**Authors:** Hardy Rolletschek, Achim Stangelmayer, Ljudmilla Borisjuk

**Affiliations:** 1 Institut für Pflanzengenetik und Kulturpflanzenforschung (IPK), Corrensstr. 3, 06466 Gatersleben, Germany; E-Mail: rollet@ipk-gatersleben.de (H.R.); 2 Presens Precision Sensing GmbH, Josef-Engert-Strasse 11, 93053 Regensburg, Germany; E-Mail: achim.stangelmayer@presens.de (A.S.)

**Keywords:** Hypoxia, microoptode, oxygen sensing, seed development, seed photosynthesis

## Abstract

Oxygen deficiency is commonplace in seeds, and limits both their development and their germination. It is, therefore, of considerable relevance to crop production. While the underlying physiological basis of seed hypoxia has been known for some time, the lack of any experimental means of measuring the global or localized oxygen concentration within the seed has hampered further progress in this research area. The development of oxygen-sensitive microsensors now offers the capability to determine the localized oxygen status within a seed, and to study its dynamic adjustment both to changes in the ambient environment, and to the seed's developmental stage. This review illustrates the use of oxygen microsensors in seed research, and presents an overview of existing data with an emphasis on crop species. Oxygen maps, both static and dynamic, should serve to increase our basic understanding of seed physiology, as well as to facilitate upcoming breeding and biotechnology-based approaches for crop improvement.

## 1. Introduction

Seed development is an essential part of plant reproduction, and plant seeds are the basis for much of human and animal nutrition. One of the major aims of plant physiology research is to elucidate the molecular mechanisms underlying seed growth, and especially, to determine what constitute its most important limiting factors. A body of indirect evidence points to the presence of oxygen-deficient zones within the seed [1-5], and these are responsible for localized partial (hypoxia) or even complete (anoxia) inhibition of mitochondrial respiration. Under the former condition, the rate of mitochondrial respiration is governed by the sub-optimal concentration of oxygen present, while under the latter, respiration ceases because of a lack of oxygen required for ATP production. Oxygen deficits within the seed can, in principle at least, affect gene expression, enzyme activity, metabolite levels and metabolic fluxes [6]. Thus they determine the pattern of growth of the developing and/or germinating seed, with knock-on effects on the level of plant fitness and survival. Assuming that endogenous hypoxia is deleterious with respect to both seed yield and germinability, its reduction or prevention could be advantageous for plant productivity. However, a strategy based on this assumption needs to be supported by a detailed knowledge of the factors determining both the extent and the topological distribution of hypoxia. Oxygen-sensitive microsensors have provided a means to directly measure localized oxygen status within the developing seed of several major crop species (soybean, pea, maize, barley, wheat, sunflower and oilseed rape), delivering a picture of internal steady-state oxygen levels with a high degree of spatial resolution. These measurements have been used to generate oxygen maps, to characterize dynamic changes in tissue oxygen levels in response to environmental factors, and to monitor the rate of endogenous release of photosynthetic oxygen. Here we review both the relevant methodology and the major outcomes of applying microsensors to plant seeds.

## 2. Short Overview on Microsensors

Two types of oxygen-sensitive microsensors are commonly used for fine scale measurements of the oxygen distribution: electrochemical or optical ones. Electrochemical oxygen microsensors, called microelectrodes, usually are miniaturised Clark-type oxygen electrodes [7]. Optical oxygen microsensors, called microoptodes, are based on fiber optic setups [8-10].

The manufacture of electrochemical oxygen microsensors is a time consuming and complex procedure. In addition, storage and transportation of these oxygen microsensors can be difficult, so availability of high quality electrochemical oxygen microsensors has always been the challenge for researchers. This encouraged scientists to develop optical oxygen microsensors [8-10], that were more easily to manufacture, that could be stored over several years without risk of oxidation, and could be transported easily. The optoelectronic measuring system for oxygen microoptodes consists of a fibre-coupler, optical filters, lenses, light source (light-emitting diode) and light detector (photodiode); a signal-processing unit (phase-angle detection, filtering) and digital signal processing (control, data storage and display). The oxygen concentration is measured with tapered glass fibres (tip diameter approximately 50 μm) by the dynamic quenching of a luminophore. A phase-modulation technique is used to determine the phase-angle shift that is caused by the fluorescence lifetime when the indicator is excited sinusoidally. Small and portable oxygen meters were developed. Microoptodes can be calibrated easily with a two point calibration. The drift of the sensor signal of oxygen microoptodes can be as low as 0.1 percent oxygen within a period of 30 days. This drift is much lower as compared to electrochemical oxygen microsensors over such long time intervals. Due to their robustness, reliability and long term stability microoptodes today are widely used in various biotechnological applications, like tissue engineering [11].

Fiber optic sensors display the following advantages over microelectrodes:

- Affordable price,
- Measures oxygen in liquid as well as in the gas phase,
- Sensor signal independent of flow velocity,
- No time for polarization required, unlike the electrochemical electrode,
- No consumption of oxygen molecules while measuring, unlike the electrode that consumes oxygen molecules,
- No cross-sensitivity and no interference to carbon dioxide (CO_2_), hydrogen sulfide (H_2_S), ammonia (NH_3_), pH, and any ionic species like sulfide, sulfate or chloride. Oxygen microoptodes are only affected by gaseous sulfur dioxide (SO_2_) and gaseous chlorine (Cl_2_),
- Measurement range from 1 ppb up to 22.5 ppm dissolved oxygen
- Fast response times (t90 up to 1 s in the liquid and < 0.2 s in the gas phase).

While there are a number of good reasons for using optical sensors, there have one disadvantage: microoptodes have a tip size of approximately 50 μm, which is relatively big as compared to microelectrodes (10 μm and less). This hampers studies where a very high spatial resolution is needed, e.g. to map gradients in oxygen concentration over a few cell layers. However, microoptodes allow spatial resolutions of slightly below 50 μm, which is sufficient for most applications.

## 3. Oxygen Mapping in Plant Seeds

Oxygen-sensitive microsensors have enjoyed a long history of use in plant biology with focus on roots and its nodules [12-15]. The first (albeit indirect) attempt on seeds was done by Porterfield *et al.* [16] using miniature glass electrodes. By assessing the endogenous oxygen status within the siliques of both thale cress (*Arabidopsis thaliana*) and oilseed rape (*Brassica napus*) it was proposed that oxygen deficiency is an important determinant of the process of seed development. A series of studies followed, in which direct estimates of endogenous oxygen concentrations were made using relatively robust oxygen probes (microoptodes; Presens GmbH Germany). The procedure for oxygen profiling in seeds has since been standardized into the following four steps:

1. The fruit (containing the intact seed) is fixed in a horizontal plane and, if necessary for the access of the microsensor, interfering material of the fruit is removed (e.g. a small window is cut into the pod wall of a leguminous species, while in maize, the husk is discarded).
2. Correct positioning of the microsensor on the seed surface is aided by a microscope. In some cases, the sealing of the microsensor entry point is necessary to prevent the diffusion of oxygen into the seed via the micro-channels formed by the probe. Often this is achieved by the application of silicone grease.
3. The microsensensor is driven, in a series of timed steps, into the seed using a micromanipulator. After each pulse, it is usual to pause for ∼ 10 s to allow equilibration and to obtain a local measurement.
4. After measurement, the seed is dissected along the measurement transect to identify each of the structurally distinct zones of the seed (seed coat, vacuole, embryo, endosperm). This is necessary to relate measurements of oxygen concentration with each physiologically and functionally distinct portion of the seed.

**Figure 1.**
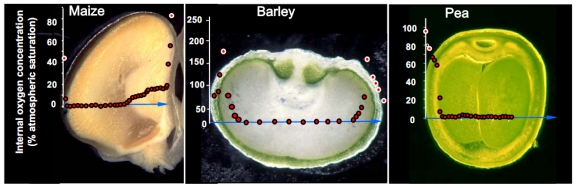
Characteristic oxygen profiles measured in seeds of maize, barley and pea (left, middle and right panel, respectively). Oxygen was measured using microsensors along the x-axis. Oxygen concentration is given in % of atmospheric saturation (100% corresponds to 258 μM at 25 °C). Please note different scales for y-axis. After measurement, seeds were cross-sectioned along the pathway of sensor penetration. The overlay of tissue structure an oxygen gradient represents the “oxygen map”. Profiles for maize and pea were measured in darkness, while in barley seeds were illuminated with 700 μmol quanta m^-2^ s^-1^. Oxygen maps were taken from [20, maize], [21, barley] and [19, pea].

Following this general procedure, oxygen maps have been created for the seeds of soybean [17], pea and broad bean [18,19], maize [20], barley [21], sunflower [22] and oilseed rape [6]. Some examples are shown in Figure 1. For wheat, only single location measurements [23], but no oxygen profile across the seed, are as yet available. Complete oxygen maps have established the reality of localized hypoxia within the seed, covering the major part of the maize and barley endosperm and the pea embryo (Figure 1).

The maps accord well with inferences based on biochemical assays and growth experiments [1-5]. Seed oxygen concentrations are developmentally regulated, and, for chlorophyll-bearing seeds, are also influenced by ambient light intensity [6]. Sensitivity to these factors is species-dependent. Moreover, microsensor-based oxygen mapping has allowed for the identification of regions having a high diffusional impedance for gas exchange - one example is the outer suberin-containing aleurone layer of the maize caryopsis, across which there is a substantial oxygen concentration gradient (Figure 1, left panel). It has further become possible to characterize certain gas diffusion pathways [20] as well as to quantify localized photosynthetically induced oxygen release [21]. The high photosynthetic activity of the pericarp chlorenchyma in the barley caryopsis allows for the build-up of significant endogenous oxygen levels in the tissue, reaching almost double the atmospheric concentration (Figure 1, middle panel). Thus, microsensor-based mapping can also identify regions which underly hyperoxic (stress) conditions which is physiologically quite different from hypoxic stress. In brief, oxygen mapping in seeds has provided a wealth of information regarding endogenous oxygen status, and has facilitated the association of oxygen gradients with specific regions of the seed.

## 4. Monitoring Environmental Effects on Steady-state Oxygen Concentrations within the Seed

**Figure 2.**
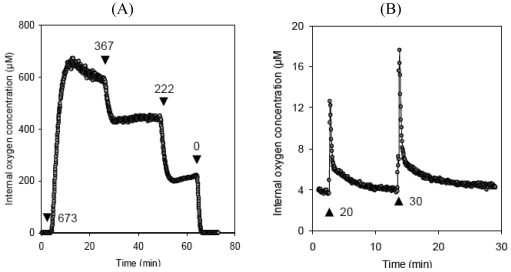
Dynamics of steady state oxygen concentration in seeds. (A) Oxygen level measured in rapeseed under distinct light conditions: in darkness, internal oxygen level is below 2 μM, but responds immediately to illumination in a dosage-dependent manner. Arrows indicate the time point of illumination, the numbers give the light intensity in μmol quanta m^-2^ s^-1^. (B) Oxygen level measured in soybean cotyledons in response to nitric oxide. After insertion of the microsensor into the seed, trace amounts of NO were injected into the intact seed using a microsyringe. Endogenous oxygen level responsed immediately to the injection of 20 and 30 pmol nitric oxide (as indicated by the arrows; for experimental details please see Borisjuk *et al.*, [26]).

Most seeds are photosynthetically active during at least some stages of development. Light availability within the seed is limited to about 10% of incident radiation (soybean [17]; white lupin [24]; oilseed rape [25], but photosynthetic activity within the seed can be responsible for rapid changes in the steady-state oxygen concentration, as has been demonstrated by microsensor-based measurement of the effect of varying ambient light conditions (for experimental details, see [26]). An illustration of the outcome of this sort of experiment is given as Figure 2A, which plots the oxygen level 1 mm below the surface of an oilseed rape seed. In the absence of light, the level is < 2 μM, but when illuminated with 673 μmol quanta m^-2^ s^-1^, it rises instantaneously to almost 700 μM, and stabilizes at ∼ 600 μM. Stepwise reductions in light intensity result in an immediate response, which settles after some delay into a new, lower steady-state level. Thus seed photosynthesis can deliver significant amounts of oxygen, which can serve to relieve localized hypoxia (turning into hyperoxia!), and allows the green seed a means to stimulate metabolism in the presence of light.

Hypoxia is associated with the endogenous production of nitric oxide (NO) [27], a known modulator of respiratory activity in both plant and animal tissues [28-30]. It has been suggested that NO can also affect the consumption, as well as the availability,of oxygen within the tissue, a proposition which has been experimentally verified in both the soybean and pea seed [26]. In these experiments, an oxygen-sensitive microsensor was inserted into the embryo of an intact seed, and trace amounts of NO were injected through a microsyringe. The oxygen concentration increased immediately after the injection, but within a few minutes, the injected NO was degraded and the initial oxygen level restored (Figure 2B). The effect was dosage dependent, i.e. the increment in oxygen level correlated positively with the quantity of NO injected – cf. 20 *vs* 30 pmol injections in Figure 2B). The reversible repression of respiration as mediated by NO is a significant component of *in vivo* regulation of cellular respiration, and is also critical to the balancing of the oxygen status of the cell/seed [26]. This example illustrates how oxygen-sensitive microsensors can facilitate the tracking of dynamic changes of a seed's oxygen status, and allow insights into its regulation in response to changes in ambient environmental conditions. Notably, instantaneous variation in both cellular respiration and photosynthesis can be monitored with a high level of accuracy. One can even achieve a degree of spatial resolution when inserting the microsensor at different depths and repeating the microinjection experiment. This would allow generation of 2-D maps showing possible tissue-specific differences in the regulatory responses – a feature which is not achievable by any other current technique.

## 5. The Establishment of Hypoxic Conditions during Seed Germination

**Figure 3.**
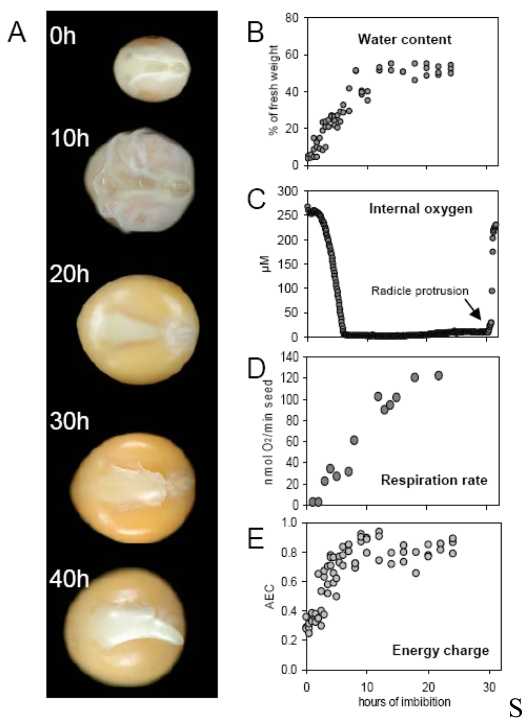
Morphological and biochemical changes during imbibition of seeds of pea (Pisum sativum L., var Baccara). (A) Visual changes of dry seeds (0 h) with swelling of seed coat (after 10 hours) and cotyledons (20 h), radicle protrusion breaking the seed coat (30 h) and radicle outgrowth (40 h). Concomitant changes were monitored for water content of the embryo (B), internal oxygen concentration (C), respiration rate (D), and adenylate energy charge (E). Data in (B-E) are taken from [36].

Seed germination and early seedling growth have been a focus of classical research exploring hypoxia in the seed [31,32]. The process of germination starts with the uptake of water (“imbibition”), and a very early event is the re-establishment of mitochondrial respiration. This is accompanied by massive changes in gene expression, which include an increase in both the transcription and enzymic activity of proteins associated with the mitochondrial TCA cycle and energy provision [33-35]. This process has been fairly well documented, but historically has not been able to consider the oxygen status of the germinating seed, and how this may interact with the germinating process. The conventional assumption is that the activation of respiration increases the oxygen demand of the seed, with likely downward pressure on the internal oxygen concentration. The use of microsensors to track oxygen status following imbibition has been reported recently [36]. A narrow hole (diameter ∼ 100 μm, length ∼ 500 μm) was drilled into a pea seed, and the tip of a microsensor was inserted into the hole, which was sealed with silicone grease to prevent any diffusive gas exchange. Once the sensor signal had stabilized, imbibition was initiated and the internal oxygen concentration was continuously monitored (Figure 3C). While the oxygen level was essentially in equilibrium with the atmosphere pre-imbibition (∼ 250 μM), within seven hours it fell rapidly to reach close to anoxia depletion (< 3 μM). This decline was related to both a lower rate of oxygen diffusion through the imbibed seed coat/tissues, and to a rise in respiration rate (see Figure 3D/E). The strong hypoxia of the imbibed pea seed has been predicted from the observed timing of the onset of fermentation [37]. When oxygen supply was increased to the imbibed seed, a clear increase in adenylate energy charge, and a corresponding decrease in the quantity of fermentation products were noted ([36] and own unpublished data).

Experimentally induced seed hyperoxia favours the growth of the pea seedling, but has little effect on its germination rate (D. Macherel, pers. comm.). Therefore, the imbibed pea seed is adapted to tolerate hypoxia, and factors other than the rate of ATP generation must be limiting for germination. The sensitivity of germination to oxygen limitation is species-dependent [31,32]. The data shown in Figure 3C also show that the rupturing of the seed coat by the emerging radicle triggers a large increase in the internal oxygen concentration. This underlines the importance of the seed coat as a barrier to gas diffusion [32].

The photosynthetic apparatus present in most dicot embryos and monocot pericarps is degraded as the seed matures, and therefore the ability of the seed to photosynthetize is lost [38]. There are only some exceptions like the sacred lotus (*Nelumbo nucifera*), in which part of the mature embryo contains chlorophyll. While photosynthetic activity is unlikely to be occurring in the dry seed, it remains unclear whether photosynthesis is initiated during early germination in the presence of light. Photosynthetic oxygen release from the embryo could serve to alleviate the characteristic hypoxia of the imbibed seed, and thereby would represent an interesting adaptive strategy for enhancing the ability to germinate in adverse environmental conditions.

## 6. Conclusions

The application of oxygen-sensitive microsensors in seed science has significantly improved our picture of the oxygen status of the seed, and our understanding of the mechanisms for oxygen balancing. The spatial resolution achieved by oxygen mapping has created novel opportunities to study both the pathways and the barriers involved in diffusional gas exchange. The experimental data gained from microsensor experiments provide a sound basis for anticipated computer-assisted approaches, in which the complex regulation of oxygen homeostasis in the seed via diffusion, respiration, photosynthesis, etc. can be modelled. A microoptode tip diameter of 50 μm has been typical for oxygen mapping in the seed till now, but miniaturization should lead to further improvement in spatial resolution. The prospect of combined applications of O_2_, CO_2_ and NO-sensitive microsensors is a particularly promising future development.
